# Recurrent Thymic Carcinoma Manifesting as a Diaphragmatic Mass: A Rare Presentation

**DOI:** 10.7759/cureus.99772

**Published:** 2025-12-21

**Authors:** Riyah Mehnaz Ayinikunnan, Gowtham Karthik V, Suhaildeen Kajamohideen, Ludia John

**Affiliations:** 1 Department of General Surgery, Sri Ramachandra Institute of Higher Education and Research, Chennai, IND; 2 Department of Surgical Oncology, Sri Ramachandra Institute of Higher Education and Research, Chennai, IND

**Keywords:** anterior mediastinum, diaphragmatic mass, mediastinal neoplasm, neoadjuvant chemotherapy, thoracic surgery, thymic carcinoma

## Abstract

Thymic carcinoma is a rare and aggressive tumor of the anterior mediastinum, known for its varied presentations and unpredictable behavior. We report a case of a gentleman in his fifties, with a prior history of thymic carcinoma, who presented with a large thoracic mass measuring nearly 20 cm, suggestive of a recurrence, yet remarkably with no evidence of distant spread. He was managed through a multidisciplinary approach and remains disease-free at the end of one year. This case highlights the locally invasive nature of thymic tumors and underscores the importance of comprehensive, team-based management in achieving favorable outcomes in such rare malignancies.

## Introduction

Thymic carcinoma is a rare and aggressive anterior mediastinal neoplasm, often discovered incidentally or through symptoms of mass effect or paraneoplastic syndrome [1]. It has an incidence of approximately 0.5 cases per million and is associated with a poor prognosis [2]. These tumors are usually locally invasive and diagnosed at advanced stages [3]. Due to these constraints and heterogeneity of cases, standardized diagnostic and treatment approaches are challenging, leading to management often being tailored on a case-by-case basis [2,3]. We present a case of non-metastatic, twice recurrent thymic carcinoma, status post complete surgical resection of the tumor.

## Case presentation

A male in his early fifties presented with complaints of pain in the left lateral chest wall and left hypochondrium, loss of appetite, and loss of weight for a year. He was a known case of thymic carcinoma, which was first found incidentally during an anesthetic workup for an orthopedic surgery in 2010 in our institution. The anterior mediastinal mass was excised, and the histopathological report revealed well-differentiated thymic carcinoma.

Upon diagnosis of thymic carcinoma, the patient sought to continue treatment at another center, where he underwent 28 cycles of radiotherapy followed by four cycles of chemotherapy with the CAPP (cyclophosphamide, doxorubicin, and cisplatin) regimen and was kept on regular follow-up with PET-CT scans yearly. Five years later, a routine PET-CT scan showed a pleura-based soft tissue density lesion in the left posterior mediastinum measuring 4.63 cm x 8.46 cm x 7.19 cm. The lesion was excised by thoracotomy, and histopathological examination of the biopsy confirmed recurrence of thymic carcinoma. Following surgery, he received six cycles of pemetrexed and carboplatin combination chemotherapy. A PET-CT after chemotherapy showed no active disease. In the years that followed, he was kept on routine follow-up every three months for the first year and every six months thereafter. He was asymptomatic until 2021, after which he started experiencing mild left lateral chest wall pain radiating to the left hypochondrium and back. A PET-CT was taken, which showed a fluorodeoxyglucose (FDG) avid soft tissue density lesion in the left posterior diaphragmatic pleura measuring 5.55 cm x 5.54 cm x 2.59 cm (maximum standardized uptake value (SUVmax) of 2.52). A second recurrence was suspected, but no surgical intervention was done, and the patient was monitored for progression of the mass using serial chest X-rays due to the financial constraints of the patient.

**Figure 1:**
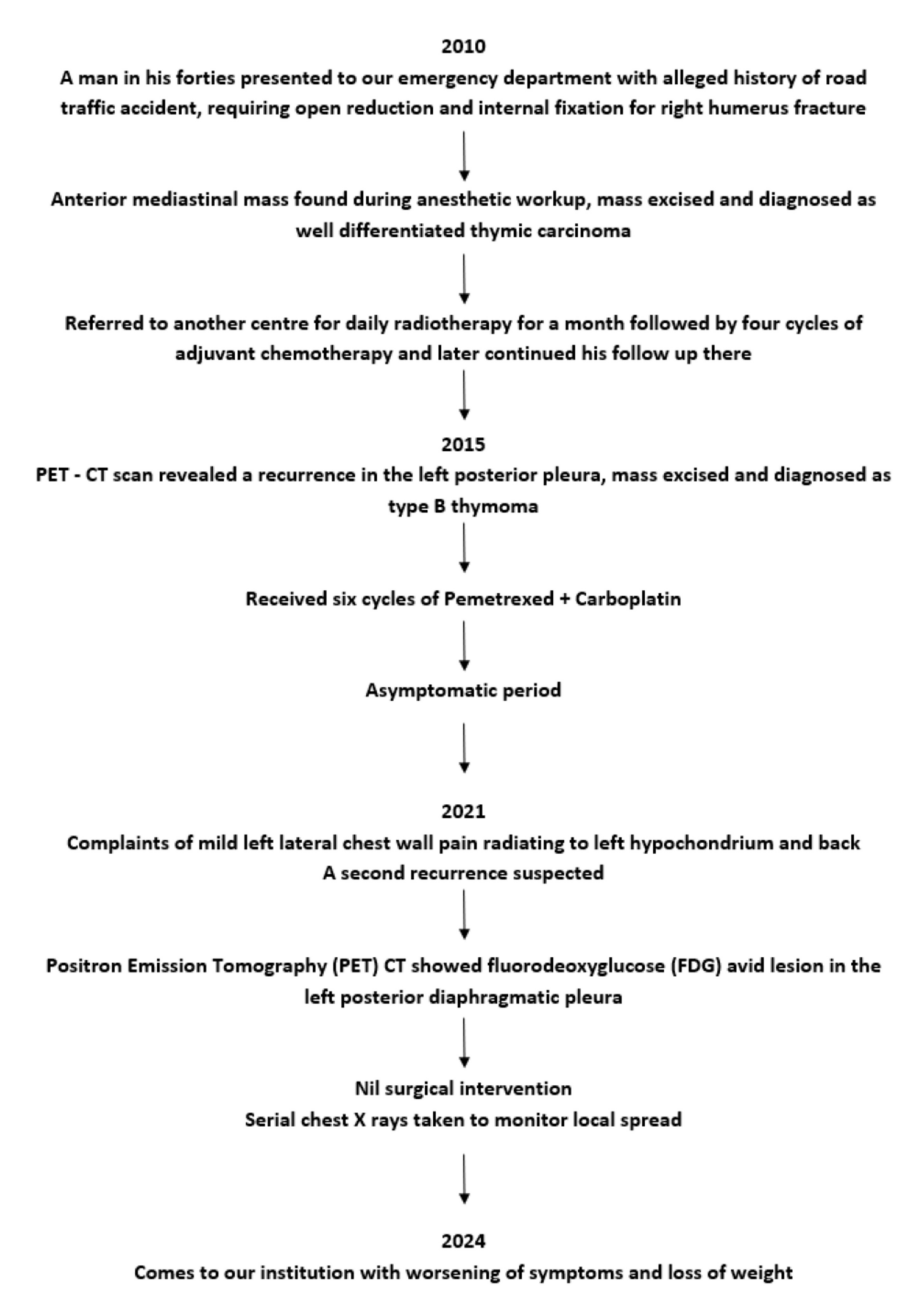
Flowchart showing the patient's clinical course

In 2024, due to worsening of symptoms and loss of 8 kg of weight in a year, he turned to our institution for further evaluation and management (Figure 1). 

Investigations

**Figure 2:**
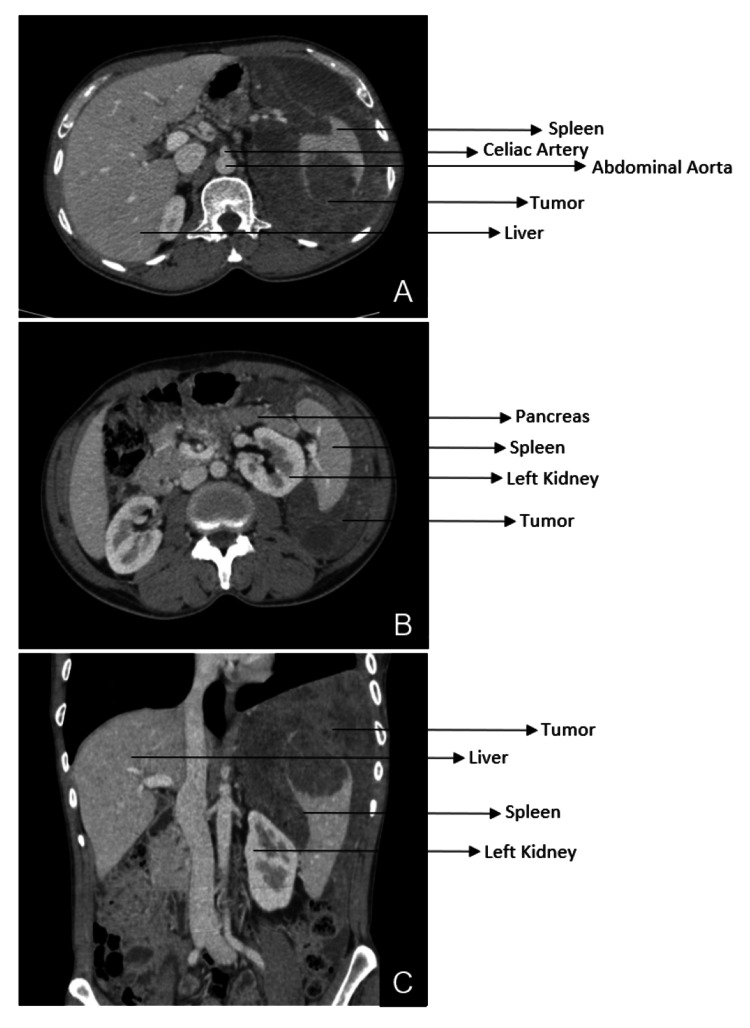
Preoperative CT scan of the thorax and abdomen with contrast (A) Axial section showing left infra-diaphragmatic soft tissue mass encasing the spleen; (B) Axial section showing left infra-diaphragmatic soft tissue mass displacing the left kidney anteromedially; (C) Coronal section showing infra-diaphragmatic extension of the tumor

The most recent CT of the thorax and upper abdomen with contrast done preoperatively showed a relatively well-defined, heterogeneously enhancing, soft tissue mass in the left subdiaphragmatic region with mild supradiaphragmatic extension measuring 21.5 x 17 x 12 cm, a significant decrease in size as compared to the previous PET-CT report (26.2 x 17.5 x 14.5 cm) done before three cycles of neoadjuvant chemotherapy. The radiological extent of the lesion was as follows. Medially, the lesion was seen insinuating into the lesser sac region with loss of fat planes with the left lobe of the liver. There was complete encasement of the splenic artery with no evidence of thrombus or luminal narrowing (Figure 2A). The lesion compressed the left adrenal gland with loss of fat planes and displaced the left kidney anteromedially with loss of fat planes in the upper pole (Figure 2B). Laterally, the mass abutted the left coastal and diaphragmatic pleura and the left lateral abdominal wall (Figure 2C). Inferiorly, the lesion was seen extending into the epigastrium, left hypochondrium, left lumbar, and left iliac regions, with the lower border of the lesion seen at the level of the L5 vertebral body. It infiltrated the superior aspect of the spleen, and there was loss of fat planes of transverse and descending colon loops (Figure 2C). Superiorly, it extended into the supradiaphragmatic region involving predominantly the left posterior pleura.

Treatment

**Figure 3:**
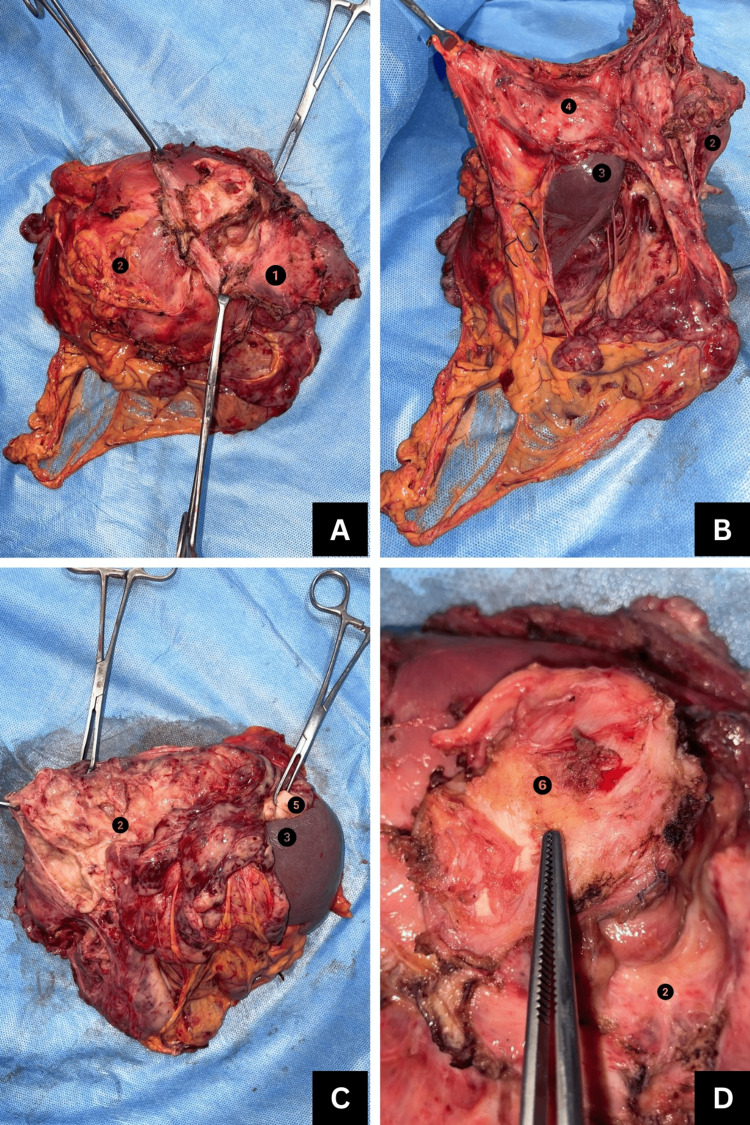
Resected tumor specimen in toto: (A) left hemidiaphragm along with tumor; (B) tumor encasing and adherent to spleen removed in toto; (C) left adrenal gland along with perinephric fat removed; (D) periosteal clearance of supradiaphragmatic extension of tumor 1: left hemidiaphragm; 2: tumor mass; 3: spleen; 4: parietal peritoneum; 5: left adrenal gland; 6: periosteum of left rib

**Figure 4:**
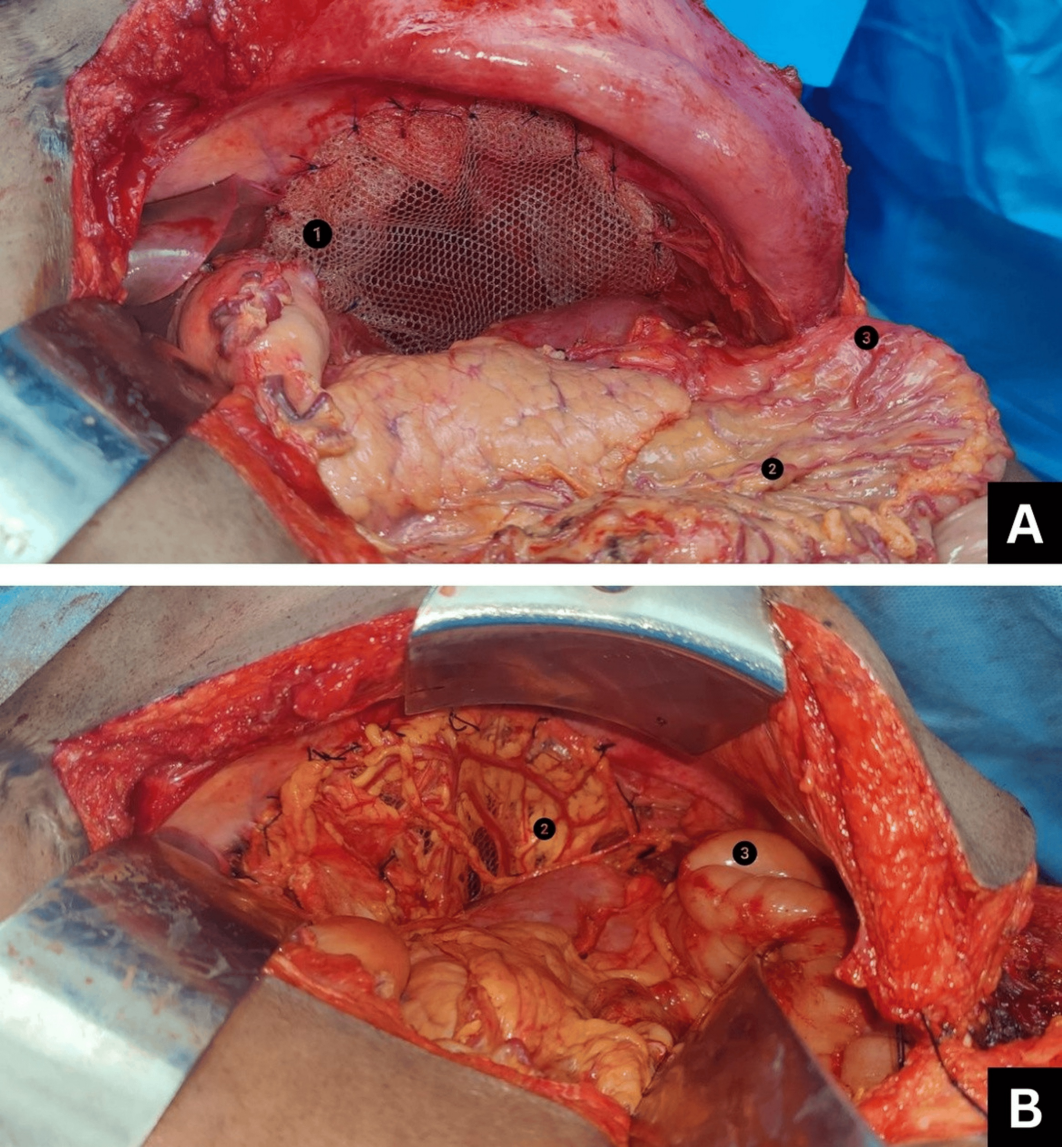
Left diaphragm reconstruction using a Prolene mesh and omentum: (A) Prolene mesh sutured in place of the left hemidiaphragm; (B) omental patch sutured over the mesh 1: Prolene mesh; 2: omentum; 3: small bowel loop

The patient received three cycles of neoadjuvant chemotherapy with doxorubicin 70 mg, cyclophosphamide 700 mg, and cisplatin 70 mg (CAP) regimen [4] with an interval of 21 days between each cycle. The tumor was reassessed using PET-CT, which showed the above-mentioned findings. Consequently, he was planned for open en bloc excision of the left diaphragmatic tumor with mesh reconstruction under general anesthesia. The plan was to approach the mass through the abdominal cavity, followed by excision of the left hemidiaphragm to enter the thoracic cavity and resect the supradiaphragmatic part of the tumor. On laparotomy, the tumor was found completely encircling the spleen and splenic vessels and adherent to the aortic hiatus and left adrenal gland. The left lung lower lobe was adherent to the left hemidiaphragm. The entire length of the colon, stomach, left lobe of the liver, pancreas, and left kidney was found free and dissected off the mass. The left hemidiaphragm, along with the tumor, spleen, left adrenal, perinephric fat, left supradiaphragmatic, and left lumbar parietal peritoneum, was removed in toto (Figure 3). The left hemidiaphragm was reconstructed using Prolene mesh (Ethicon, Inc., Somerville, NJ) and omentum (Figure 4). Immunohistochemistry positivity for P40 and P63 tumor markers and histopathological examination of the final biopsy confirmed thymic carcinoma, favoring squamous cell carcinoma. The patient was stage III according to the Masaoka-Koga Staging System [5]. He received three cycles of adjuvant chemotherapy with the CAP regimen. A repeat PET-CT scan showed no residue in the diaphragmatic region or distant metastasis. Following this, he underwent adjuvant radiotherapy; 4500 cGy was delivered to the thorax and abdomen in 25 fractions of 180 cGy each for a total of 25 days, five days a week.

Outcome and follow-up

PET-CT demonstrated no evidence of disease at the end of one year post treatment. He remains asymptomatic and is under regular surveillance at six-month intervals.

## Discussion

The anterior mediastinum is the most common location for mediastinal masses in adults [1]. The common masses found here are often referred to as the “terrible T’s”, namely, thymoma, teratoma, “terrible” lymphoma, and thyroid tissue. Thymic lesions account for one-half of all anterior mediastinal masses [6], yet the incidence of thymic carcinoma is between 0.07 and 0.38 cases per 100,000 person-years [7], making it an extremely rare tumor.

Thymic carcinomas are locally aggressive and often present as large infiltrative lesions with symptoms of mass effect on surrounding mediastinal structures. They also have a high propensity for early relapse and distant spread to the lung, bone, brain, and liver [8]. Huang compared patterns of relapse in thymic carcinoma and thymoma and corroborated that 60% of thymic cancer relapses occurred at distant sites [6,8]. In contrast to this, our patient experienced an unexpected clinical course with two recurrences and no distant spread. The recurrent lesions in our case were local and contiguous with the primary tumor bed rather than new or distant metastases. With the tumor spanning almost 20 cm, which typically suggests distant spread, PET-CT surprisingly showed no signs of metastasis.

Initial diagnostic evaluation of thymic tumors can be done by CT or MRI, though a recent study has revealed the latter to be superior [9]. Intrathoracic and extrathoracic spread should be evaluated preoperatively by PET-CT rather than just CT, as it can detect lymph nodes, pleural seeding, and adjacent structure invasion with more sensitivity [10]. Studies done by Yon Mi Sung and Julie Hephzibah have also tried to assess the usefulness of PET-CT to distinguish between high-risk thymomas and thymic carcinomas; however, its ability to differentiate histologic differences is not fully established [10,11]. With the technological advancements in high-resolution cross-sectional imaging, we insist that thymic carcinomas be monitored for disease progression and treatment outcomes with serial CT scans rather than chest X-rays, as was done in the case of this gentleman at his previous hospital, leading to a delay in treatment.

This case report also emphasizes the need for a multidisciplinary approach given the heterogeneous, rare, and aggressive nature of thymic carcinoma. Despite the lack of universal standardized protocols, the expert consensus is complete surgical resection whenever feasible, supported by perioperative chemoradiotherapy [6,12,13]. Given the radiosensitive nature of the tumor, ongoing technological advancements are likely to expand the role of radiotherapy in achieving favorable clinical outcomes [3]. Over the past decade, parallel advances in high-quality cross-sectional imaging, surgical approaches, and systemic therapies have augmented patient prognosis, which further reiterates the belief that a more comprehensive treatment plan is better than monotherapy [13,14].

In conclusion, large, multi-institutional cohort studies are required to further enhance the standardization of diagnosis and management of thymic tumors. This case underscores the importance of advanced imaging for accurate staging and follow-up and highlights the need for an individualized, multimodal treatment approach to improve patient outcomes in this rare and aggressive disease.

## Conclusions

Thymic carcinoma poses a significant therapeutic challenge due to its aggressive course and high rates of recurrence. Large thymic tumors can recur without distant metastasis. This case reiterates that vigilant long-term surveillance with the right imaging modality remains key to improving prognosis in such rare malignancies. A favorable outcome can be achieved despite multiple recurrences with careful planning and multimodal therapy in the absence of standardized universal treatment protocols.
